# Spinal Cord Stimulation for Chronic Pelvic Pain Secondary to Oncologic Complications: A Case Report

**DOI:** 10.7759/cureus.105136

**Published:** 2026-03-12

**Authors:** Nandini Sojitra, Ruchir Gupta

**Affiliations:** 1 Pain Management, Mountain View Headache and Spine Institute, Phoenix, USA

**Keywords:** cancer-related pain, chronic pelvic pain, multi-organ cancer, neuromodulation, spinal cord stimulation

## Abstract

This case report presents the efficacy of spinal cord stimulation (SCS) in a patient with chronic, treatment-resistant pelvic pain secondary to oncologic complications and associated therapies. A 62-year-old male with a complex history of colorectal and prostate cancer, with metastatic involvement of the bladder and breasts, presented with severe, diffuse, generalized pelvic pain rated 10/10 in severity. The patient’s pelvic pain was likely multifactorial in etiology, consisting of mixed neuropathic and nociceptive pain from extensive chemotherapy, radiation therapy, and surgery. He tried multiple conservative treatments, including opioids, IV ketamine infusions, and ganglion impar blocks, which provided only transient analgesia. The patient then underwent SCS implantation. After the procedure, he reported moderate improvement in his previously severe pre-procedural pain and continues to report sustained analgesia at his monthly follow-up visits.

## Introduction

Spinal cord stimulation (SCS) is a treatment modality used to address multiple etiologies of chronic pain, such as radicular spinal pain, complex regional pain syndrome, failed back surgery syndrome, and visceral abdominal pain. During the SCS implant procedure, electrodes are placed in the posterior epidural space and steered to the appropriate location, where they can provide electrical impulses that interfere with ascending pain signals [1]. It is hypothesized that pain impulses carried by slow-conducting C fibers and A-delta fibers can be disrupted by stimulating fast-conducting A-beta fibers, which provide inhibitory modulation of pain [1]. Activating the A-beta fibers through SCS can attenuate the perception of pain [1]. Pain management with SCS may be indicated when patients have failed multiple conservative treatment options, such as pharmacologic therapy, physical therapy, radiofrequency ablations, and nerve blocks [1]. SCS has been documented to alleviate treatment-resistant chronic pain in many patients, including those with an extensive oncologic history who may be experiencing pain post-chemotherapy and radiation therapy [2]. SCS is traditionally used to address diffuse symptoms, such as peripheral neuropathy and limb pain, and it is less frequently used to target specific pain, such as pelvic pain, unilateral limb pain, and mononeuropathies, since it is more technically challenging to reach certain pain dermatomes [1]. This case report highlights that SCS may be an effective treatment option for targeting chronic pelvic pain resulting from anti-cancer therapies.

## Case presentation

A 62-year-old male with a complex history of multi-organ cancer presented with severe, diffuse, generalized pelvic pain rated 10/10 in severity. He described persistent, non-localized pain that impacts his quality of life. He denied any associated numbness or tingling. His symptoms were refractory to conservative pain management, including trials of oxycodone and tramadol, which provided minimal benefit. His medical history is remarkable for multiple primary and metastatic malignancies, including colorectal, rectal, prostate, bladder, and metastatic breast involvement. He had previously undergone colectomy and proctectomy with colostomy bag placement for colorectal cancer, followed by bilateral mastectomy for metastasis to the breasts. Subsequent progression of cancer to the genitourinary system led to surgical removal of the bladder and prostate. His treatment plan also included chemotherapy and radiation therapy.

The patient’s pain was likely multifactorial in origin, given his extensive surgical history and exposure to chemotherapy and radiation therapy. Given the extent of chronic pain, his initial treatment plan consisted of IV ketamine infusion. Following six IV ketamine infusion treatments, the patient continued to endorse chronic pain and reported exacerbation of pelvic and perineal pain. He was then recommended for a ganglion impar block. The patient underwent two trials of ganglion impar block, which resulted in greater than 80% reduction in pain but provided only temporary relief. Other treatment interventions, such as a hypogastric block or neurolysis, were not considered because they require repetition for sustained analgesia, and the patient was interested in a more long-term solution.

Since conservative treatment options, including IV ketamine infusion and ganglion impar block, did not provide long-term analgesia for his chronic pain, the patient was recommended for a percutaneous SCS trial, with subsequent SCS implantation if a greater than 50% reduction in pain was achieved.

**Figure 1:**
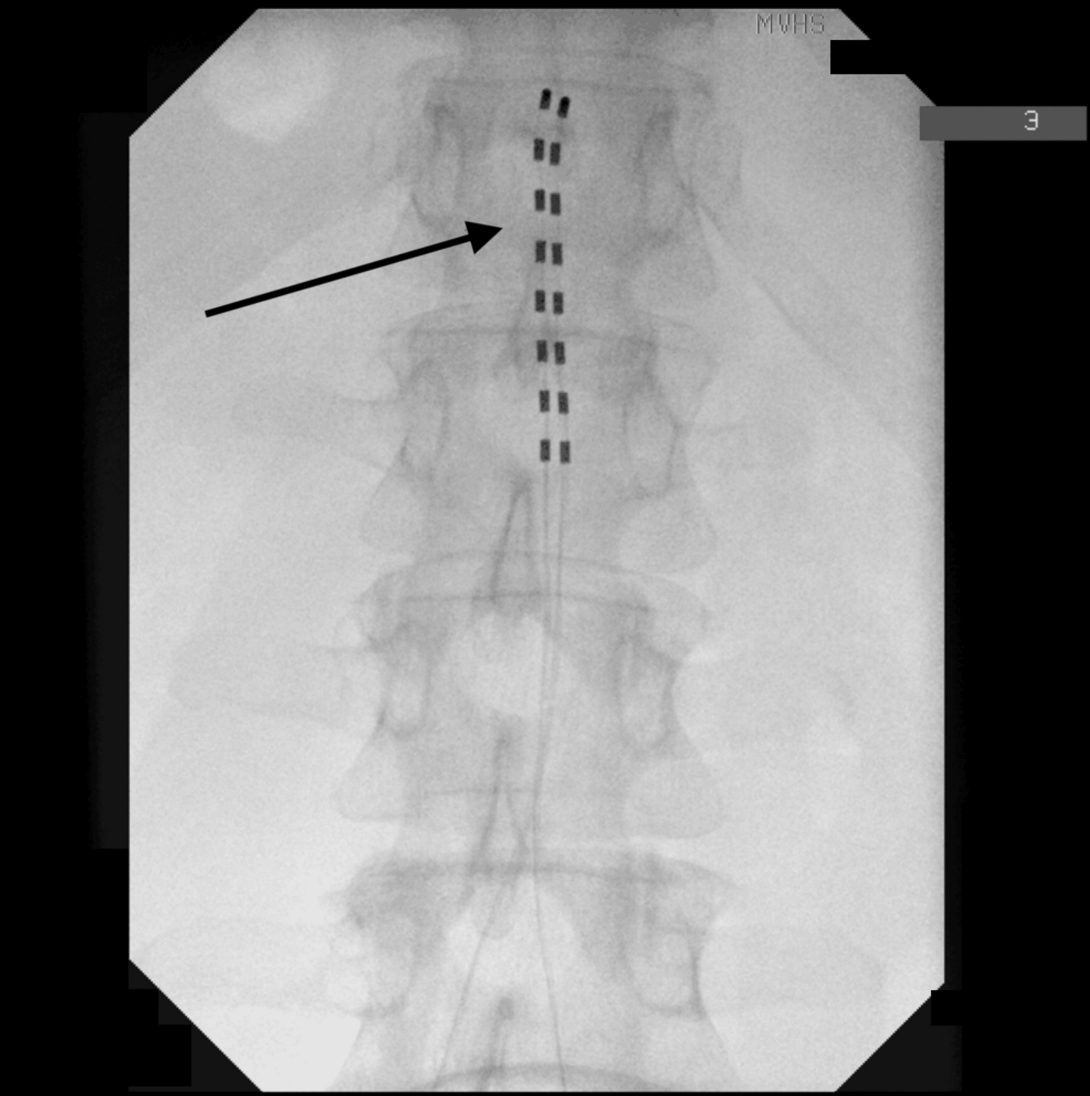
Fluoroscopy image showing spinal cord stimulator lead placement

At the one-week follow-up appointment after the SCS trial, he reported a greater than 80% reduction in his pre-procedure pain. The patient elected to proceed with SCS implantation. Percutaneous leads with electrodes were introduced at the L2-L3 level and directed to the top of T12 to achieve maximum analgesia. The intraoperative fluoroscopy image demonstrates the lead placement (Figure 1). At his one-month follow-up visit, he reported a moderate decrease in his pre-procedure chronic pain. He continues with monthly follow-up visits and reports sustained analgesia.

## Discussion

Chronic pain syndromes associated with cancer-related pain can be attributed to direct effects from neoplasm invasion and complications from chemotherapy and radiation therapy. Many cancer patients experience chronic pain that directly impacts their quality of life and overall functional capacity. The long-term goals for management of cancer pain should focus on minimizing suffering, enabling continued oncologic therapy, and improving functional capacity [1]. Treatment should be individualized, taking into account preexisting pain, psychiatric comorbidities, and other contributing factors. In the setting of chronic treatment-resistant pain, SCS has proven to be a viable therapy to provide sustained analgesia.

In this case report, the patient reported chronic pelvic pain secondary to oncologic complications and subsequent treatment, including surgery, chemotherapy, and radiation therapy. The pathophysiology of pelvic pain can be divided into three categories: nociceptive, neuropathic, or mixed pain [3]. Nociceptive pain can result from damage to superficial structures or visceral organs due to tumor infiltration of surrounding tissues and prior radiation therapy [3]. Neuropathic pain can be caused by injury to the lumbosacral plexus. Involvement of the upper part of the plexus (L1-L4) can lead to pain in the lower back, lower abdomen, and iliac crest; similarly, injury to the lower part of the plexus (L4-S3) can lead to pain in the buttocks and perineum, with referred pain to the posterolateral thigh and leg [3]. Patients can also experience nociplastic pain, which is defined as an altered perception of pain without evidence of tissue damage [4]. Extensive chemotherapy may cause nerve sensitization, which leads to hyperalgesia when nociceptors are stimulated [4]. In most cases, the pain experienced by cancer patients is typically mixed in etiology.

SCS has the potential to provide long-term pain relief from intractable pain through electrical stimulation of the dorsal spinal cord, which disrupts the transmission of ascending pain signals [2]. The FDA indications for SCS include failed back surgery syndrome, chronic painful peripheral neuropathy, multiple sclerosis, and complex regional pain syndromes I and II [5].

Though not widely used for pelvic pain, studies have shown the efficacy of this treatment modality for pelvic pain. Tate et al. conducted a prospective study that aimed to evaluate the efficacy of 10 kHz SCS in patients with chronic pelvic pain of varying etiologies, such as post-surgical complications, postpartum complications, and interstitial cystitis [6]. A total of 23 patients were enrolled, of whom 21 underwent a seven-day SCS trial. Seventeen patients (81%) reported greater than 40% pain reduction, which was the parameter set for a successful trial [6]. Thirteen of those patients subsequently proceeded with spinal cord stimulator implantation and were followed for a 12-month period [6]. The efficacy of the implants was assessed using mean Visual Analog Scale (VAS) scores for pain reduction and mean Pain Disability Index (PDI) scores for improvement in disability [6]. Patients experienced an overall 72% reduction in VAS scores and a 29-point reduction in mean PDI scores over the 12-month period [6]. The improvement in disability was clinically meaningful in 11 of 13 patients [6]. This study demonstrates that SCS can be an effective treatment option for patients with chronic pelvic pain.

The management of cancer-related pain presents a unique challenge due to its complex and multifactorial etiology. Pain in cancer patients may arise from disease progression, surgical interventions, chemotherapy, radiation therapy, or preexisting comorbidities that exacerbate pain. A systematic literature review reported that 59% of patients experienced pain following anticancer treatment, and 33% continued to report pain after curative treatment [7]. Accurate classification of the pain type, intensity, and chronicity is essential for determining an effective treatment regimen. In patients with persistent pain despite first- and second-line treatments, such as opioids, neurolytic injections, steroid injections, and radiofrequency ablations, SCS may offer a viable alternative. A retrospective study by Crowther et al. evaluated the outcomes of 77 patients who underwent SCS implantation, of whom 51 had cancer-related pain, and 22 had non-cancer-related pain [8]. The trial success rates were comparable between the two groups (73% and 77%, respectively); however, patients with cancer-related pain demonstrated a significant reduction in pain scores one year post-implantation [8]. These findings support the efficacy of SCS as an alternative therapy for managing refractory cancer pain, particularly in patients who have not achieved adequate analgesia from conventional therapies.

The decision to proceed with SCS implantation for the patient described in this case report was clinically justified, given the chronic, treatment-resistant nature of his pelvic pain secondary to oncologic complications. Despite multiple interventions, such as opioids, ganglion impar block, and ketamine infusions, the patient continued to experience debilitating pelvic pain, consistent with mixed nociceptive and neuropathic pain observed in cancer patients. In this case, SCS implantation offered a mechanistically distinct and minimally invasive approach to long-term analgesia. Intrathecal opioid therapy, which is commonly indicated for mixed cancer pain, was not considered a treatment intervention because it requires frequent refilling and is dependent on pharmacologic agents. The benefit of SCS is that it does not rely on the same receptors traditionally targeted for cancer pain (i.e., opioids) and can function 24 hours a day without the need for refilling. Furthermore, evidence from the retrospective study by Crowther et al. demonstrated a clinically meaningful reduction in pain scores and an increase in functional capacity after SCS implantation [8]. SCS was considered a rational and evidence-based therapeutic option that aligned with the broader goals of cancer pain management.

Long-term use of SCS for pain management is associated with potential complications, though these are exceedingly rare. Complications such as lead migration and infection/bleeding have occurrence rates of 1.2% and 1.9%, respectively [9]. For patients with cancer, the life expectancy of the SCS battery should be considered, as these systems often require battery exchange every 10 years, which may or may not fall within the patient’s expected lifespan.

The interpretation of the results of this case report is limited due to the single-patient study design, limited generalizability, and lack of standardized outcome measures. The main objective of this case report is to highlight an instance in which SCS provided successful pain reduction in a patient with treatment-refractory chronic pelvic pain; however, this finding cannot be generalized to a larger population of patients with similar symptoms. Additionally, the outcomes are reported as a qualitative description by the patient, which may limit the ability to determine the clinical significance of the results. The absence of a placebo limits this case report’s ability to attribute the patient’s sustained analgesia solely to the treatment intervention. Larger-scale prospective or retrospective studies could provide substantial evidence to support the efficacy of SCS in treating chronic pelvic pain.

## Conclusions

This case provides anecdotal evidence that SCS could be considered an effective treatment modality for patients with chronic cancer-related pelvic pain, specifically those who have not responded well to conventional therapies, such as pharmacologic management and interventional therapies. Individualized patient selection, comprehensive understanding of the patient’s pain etiology, and thorough assessment of pain control during the trial phase are critical factors for achieving successful outcomes with SCS therapy. The findings of this case report would be further strengthened by additional evidence from larger-scale studies.
